# Transforming community health advocates into peer interventionists: a binational harm reduction model

**DOI:** 10.3389/fpubh.2026.1811496

**Published:** 2026-09-04

**Authors:** Julia Lechuga, Rebeca Ramos, Gilberto Perez, Maria Elena Ramos, Julian Rojas, Luisa Ramos, João Ferreira-Pinto

**Affiliations:** 1 Division of Prevention Science, Department of Medicine, The University of California, San Francisco, San Francisco, CA, United States; 2 Programa Compañeros, Ciudad Juarez, Mexico; 3 College of Health Sciences, The University of Texas at El Paso, El Paso, TX, United States

**Keywords:** community health advocates, harm reduction, HIV prevention, peer interventionists, people who use drugs

## Abstract

**Background:**

Community Health Advocates (CHAs) are widely employed as outreach workers for people who use drugs (PWUD), yet there is limited evidence on how to design capacity-building interventions that strengthen peer-driven behavior change in binational border settings. This study examined a structured capacity-building model that trained peer CHAs in Ciudad Juárez, Mexico, and El Paso, Texas to deliver an HIV prevention and skills-building intervention to PWUD and assessed its impact on HIV risk and protective behaviors.

**Methods:**

We employed two complementary evaluation methods: (1) a quantitative survey with participants, and (2) in-depth interviews with CHAs. Quantitatively, generalized linear models estimated effects of intervention participation on condom procurement, procurement of sanitized syringes, and condomless sex with substance-using partners, followed by a path model testing whether receipt of HIV prevention information mediated the association between peer-attributed behavior change and condomless sex. Qualitatively, in-depth semi-structured interviews with CHAs, analyzed using a rapid qualitative approach, explored how CHAs experienced training and evolving roles, and how they perceived peer communication and information diffusion as mechanisms of change.

**Results:**

CHAs assumed expanded responsibilities, including delivering rapid HIV testing, organizing community events targeting structural determinants of HIV risk, and implementing peer-driven, network-based behavioral interventions; over time, they evolved into peer navigators and recovery coaches, facilitating linkage to harm reduction, HIV care, and overdose prevention while maintaining cultural and linguistic congruence with binational PWUD communities. Higher levels of peer-attributed behavior change were strongly associated with receiving a broader range of HIV prevention information topics, and greater receipt of information was associated with lower levels of condomless sex. Peer-attributed behavior change exerted a significant indirect effect through receipt of information, while qualitative data highlighted how trust, lived experience, and incremental practice changes supported these pathways.

**Conclusion:**

A structured, culturally attuned capacity-building model can transform CHAs into peer interventionists, navigators, and recovery coaches who deliver network-based HIV and overdose prevention strategies in binational settings. Peer-attributed behavior change appears to reduce condomless sex primarily by increasing exposure to HIV prevention information, underscoring information diffusion through peer networks as a key mechanism for lowering HIV-related sexual risk in marginalized cross-border communities.

## 1 Introduction

Community Health Advocates (CHAs) have served as trusted bridges for populations that may not access formal healthcare, especially in regions such as the U S-Mexico border affected by poverty, migration, and structural violence (1–4). Ciudad Juárez, Mexico and El Paso, Texas, sister cities on the US-Mexico border, constitute a contiguous binational metropolitan area, with city centers 3–5 km apart and a significant daily cross-border flow for work, family, and services. Many community members and CHAs maintain connections on both sides. Because HIV and other drug use health risks are concentrated in interconnected networks, drug-using networks frequently extend across the border, highlighting the importance of binational coordination for infectious disease prevention.

Along the U. S.–Mexico border, social determinants of health are compounded by high substance use, HIV risk, and a lack of culturally tailored healthcare, emphasizing the need for peer-led, community-based approaches (5–7). The US-Mexico border is a drug transshipment corridor, and cocaine and crack are common among polysubstance users and injection routes (8). There are an estimated 6,000 people who inject drugs, indicating a large population at risk for HIV (9). Research on drug-use patterns along the border shows frequent use of cocaine or crack, alone or combined with heroin (10). In this context, crack-using networks are a key focus for HIV prevention and harm reduction strategies.

Recent estimates show an HIV prevalence of roughly 416 per 100,000 El Paso residents, with new diagnoses rising by about 32% from 2019 to 2023 and nearly 90% of cases among Latine^1^ residents (11). On the Mexican side, studies in Ciudad Juárez report HIV prevalence from 3% among injection drug users to 8% among female sex workers, increasing to 12% among female sex workers who inject drugs (12). These data highlight a concentrated HIV burden among vulnerable groups and underscore the importance of implementing culturally grounded, peer-led prevention strategies in this region (13).

While peer CHAs and other community health workers have proven effective in health education, navigation, and HIV prevention, most initiatives focus on outreach or linkage roles rather than central behavioral-health intervention. Few studies detail how CHAs are recruited, trained, supervised, or supported in delivering structured, evidence-based interventions for harm reduction and behavior change, especially in binational contexts. There is limited understanding of expanding CHA roles to include advanced techniques like motivational interviewing or crisis de-escalation, or of their function in areas with criminalization, cross-border mobility, and fragmented health systems. This contributes to a gap between calls for community-led harm-reduction and practical strategies for building CHA capacity in high-risk environments.

Project Encuentro is a binational, community-based program that trains peer CHAs to provide behavioral interventions to people who use drugs (PWUD) across both sides of the El Paso-Ciudad Juárez border. Developed in partnership with local organizations and a Mexican NGO experienced in harm reduction (14), the project adapts an HIV prevention model to address local risks shaped by economic marginalization, drug-related violence, and differing policy environments. Trained CHAs and peer leaders deliver a structured, theory-based group program to drug-using networks, aiming to change group norms and reduce HIV and drug-related risks as part of a wider community mobilization effort.

This manuscript examines the development and support of CHA competencies for delivering this peer-driven behavioral intervention, how their involvement influenced HIV sexual risk behaviors, and how they grew as interventionists themselves (15–18). In doing so, the study addresses important research gaps by: (1) moving beyond descriptive accounts of CHA outreach to present an empirically based model of CHAs as behavioral-health interventionists (19–22); (2) describing a binational implementation strategy that combines community-based harm reduction organizations with academic partners (13, 15–18); and (3) demonstrating how expanded CHA skills can be used to respond to intertwined crises of poverty, migration, violence, and drug use (13, 15–17, 20–23).

The innovation of this work lies in positioning CHA-delivered interventions within a community-based participatory research (CBPR) and human-rights-based approach (24), adding to emerging efforts that view CHAs as multi-level change agents capable of impacting individual behavior, social networks, and the broader risk environment (1–4, 20–23). Ultimately, the study offers a replicable guide for expanding peer-driven, community-centered harm-reduction strategies in complex transborder areas where traditional service models often fall short for those at highest risk (13, 15–17, 20–22).

## 2 Methods

### 2.1 Study design

We employed two complementary evaluation methods: (1) a survey with intervention participants, and (2) in-depth interviews with CHAs to examine their training experiences, role development, and perceived factors affecting participant behavior change. The peer-led behavioral intervention took place from 2016 to 2022 in the cities of Ciudad Juarez, Mexico, and El Paso, Texas. It was delivered by CHA, who were peers. The intervention aimed to promote harm reduction and HIV prevention sessions among people who use drugs (PWUD) in the El Paso–Ciudad Juárez border region. The study used a community-based participatory research (CBPR) framework, highlighting equal collaboration between academic researchers and community partners throughout all stages of implementation. For example, the intervention, assessments, and implementation logistics were refined by a community advisory board (CAB). This board included members from local social, health, and substance use prevention and treatment organizations, peer CHAs, individuals with lived experience of drug use, and academic partners. The CAB met bi-monthly during the study period to review and modify survey questions, enhance intervention activities like role-plays and group discussions, and develop CHA training content and supervision. This approach helped create a culturally responsive, community-driven intervention that was tailored to the social, cultural, and epidemiological realities of the El Paso–Ciudad Juárez border area. Meaningful community-based participatory research necessitates a dedicated timeframe for establishing relationships, maintaining presence, and responding to community priorities. Such efforts are often misaligned with traditional academic schedules, funding cycles, and institutional norms. In this binational and unstable context, building trust was essential and demanded that academic partners collaborate with humility, stay actively involved, and support community members and partner organizations to steer priorities, pace, and implementation strategies.

### 2.2 Study setting and partners

The intervention took place in El Paso, Texas, United States, and Ciudad Juárez, Chihuahua, Mexico — twin cities forming a large, interconnected binational metropolitan area. The study was led by a tri-institutional partnership: The University of Texas at El Paso (UTEP), which managed research design, evaluation, and overall oversight of CHA training and data collection; Programa Compañeros (PC), a nongovernmental organization in Ciudad Juárez, which handled CHA recruitment, training, supervision, and intervention delivery in Mexico; and The Alliance of Border Collaboratives (ABC), a community-based organization in El Paso, which coordinated CHA recruitment, community outreach, and logistical support in Texas. These partners collaboratively refined the intervention’s content, training structure, and implementation procedures through ongoing CBPR-informed activities.

### 2.3 Peer CHA recruitment and selection

We recruited peer CHAs aged 18 years and older who had prior lived experience with HIV and/or drug use. These peer CHAs were recruited internally from individuals already involved in activities with partner organizations. The CHA team comprised peers from Ciudad Juárez and El Paso, chosen for their firsthand experience with drug use and their strong ties to cross-border networks. Training CHAs in both locations aimed to facilitate coordinated outreach and referral systems that accommodate participants’ mobility and cross-border risk settings. Study staff identified potential candidates who showed reliability, consistent participation, and alignment with the project’s values, including harm-reduction principles and a nonjudgmental attitude toward PWUD. Eligible candidates needed to have strong community ties and familiarity with neighborhoods and social networks most impacted by HIV and drug use in both El Paso and Ciudad Juárez. Eligibility for Peer CHAs required having lived experience of substance use and being in recovery at the time of recruitment. This ensured CHAs could leverage relevant experience and had enough stability for training, outreach, and group facilitation. The criterion was not meant to define recovery as fixed or linear but to support peers who might encounter substance-use cues, participant crises, or stress. Continuous supervision and referral options to substance-use treatment were available if CHAs relapsed or needed extra help. Those who disclosed their lived experience with HIV and/or drug use were given priority for recruitment as peer interventionists, as they could better build rapport, demonstrate behavior change, and navigate local drug-use environments. A total of four peer CHAs were selected and trained across both sites, with two based in El Paso and two in Ciudad Juárez.

### 2.4 CHA training and supervision

CHA selection and training were explicitly based on authenticity, empathy, and community trust. Desired attributes included in-depth knowledge of local drug-use environments and harm-reduction practices, empathy and nonjudgmental attitudes often shaped by personal experience with drug use and/or HIV, the ability to communicate evidence-based information in accessible, colloquial language, and a commitment to respectful, collaborative problem-solving. Training combined classroom instruction, experiential learning, and supervised practice: CHAs first attended a three-day intensive workshop covering core concepts in harm reduction, HIV and HCV transmission and prevention, and overdose response; foundational theories such as behavior change, motivational interviewing, and peer modeling; facilitation skills for small-group, peer-led sessions, including managing group dynamics, handling disclosures, and responding to distress; and the practical use of the structured curriculum, activity guides, and fidelity checklists. After this initial workshop, CHAs participated in approximately two months of on-the-job training, during which they co-facilitated sessions with more experienced staff, received real-time feedback, and joined weekly debriefings with Project Coordinators to review challenges, troubleshoot difficult situations, and ensure fidelity to the intervention protocol. Training materials included a bilingual slide deck summarizing the evidence supporting harm reduction, scripted role-play and motivational-interviewing scenarios, pocket-sized harm-reduction and referral guides, and comprehensive bilingual manuals for CHAs and clients, along with ongoing technical assistance, booster trainings, and feedback sessions aimed at maintaining skill development and intervention quality over time.

Peer CHAs were officially employed and compensated through the community partner organization, commensurate with local pay rates, and the organization has a long-standing dedication to hiring and integrating peers with lived experience into its staff. Their involvement went beyond short-term project tasks: CHAs received support to develop leadership skills and take on significant organizational roles. For instance, one of the peers hired for the study is now the president of Redumex (The Mexican Harm Reduction Alliance), whose nomination the partner organization in Mexico supported. The organization’s leadership actively participated in CHA training and ongoing supervision, offering guidance, debriefing, and problem-solving assistance as CHAs carried out intervention and outreach efforts. When CHAs faced relapse or other substance-use issues, leadership provided supportive referrals to treatment services.

### 2.5 Structure and content of intervention

The intervention was informed by Social Identity (25, 26) and Social Influence theories (27, 28) and implemented within a diffusion-of-innovations, network-based framework. We hypothesized that exposure to peer-led sessions would increase HIV-related information, strengthen motivation, and build behavioral skills (e.g., condom negotiation), while also shifting perceived peer norms through prevention discussions and visible enactment of safer behaviors. In this model, peer communication about prevention behaviors function as mediators that transmit the intervention’s influence from individual CHAs to their broader social networks, ultimately contributing to reductions in condomless sex. Since crack use often happens in sexual and transactional situations marked by power imbalances and disinhibition, the intervention focused on decreasing condomless sex. It combined harm-reduction skills development for condom negotiation and planning safer sex in environments where crack is commonly used.

The intervention consisted of three consecutive daily two-hour sessions delivered to intact social networks of five to six individuals who used drugs together. Groups were facilitated by peers in recovery who were highly trusted and well connected within local drug-using networks and who were trained to recruit eligible participants and to deliver the curriculum. Participants were eligible if they were 18 or older, reported injection drug use in the past 30 days, lived in or frequently visited the El Paso–Ciudad Juárez border area, and could give informed consent. Recruitment was done through the community partner’s harm-reduction programs, referrals from service providers and community members, and outreach by Peer CHAs. Some participants were already known to Peer CHAs through previous contact, shared networks, or the partner organization, but familiarity was not necessary, participation was voluntary. The sample was a convenience group reached via community networks and services. Recruitment faced challenges typical of a vulnerable border setting, such as housing instability, border crossings, changing contact info, survival priorities, and concerns about stigma, criminalization, and violence. These factors sometimes impacted enrollment and attendance but also highlighted the importance of peer-led and community-based recruitment. Sessions were held at partner organization sites, where staff verified eligibility, obtained informed consent, and provided participants with workbooks containing structured activities. The workbooks are included as supplementary material.

The intervention targeted sexual risk associated with crack use by addressing both individual behaviors and interpersonal processes, with Session 1 focusing on social contexts of risk and harm-reduction strategies through discussion and role plays; Session 2 addressing HIV and sexually transmitted infections using interactive exercises and skills practice for condom negotiation in high-risk situations, including transactional sex; and Session 3 emphasizing sexual rights, advocacy, and planning through the development of individualized and network-level HIV risk-reduction plans that identified three feasible actions to reduce sexual risk and support peers. Across sessions, CHAs delivered didactic information alongside interactive activities, such as discussions, role-plays, and problem-solving related to daily risk scenarios. Activities included discussions about the continuum of behaviors that would reduce substance use harm, demonstrations of condom placement, role-plays to practice assertive conversation about harm reduction strategies within networks, such as HIV testing, refusing to share syringes, condom use negotiation, and planning to utilize local syringe distribution services to boost sanitized syringe procurement and use.

Participants received lunch prior to each session and $25 for each session with a $15 bonus for completing all sessions. Peer facilitators received training in curriculum content, psychosocial behavior-change theory, small-group facilitation, HIV and sexual risk-reduction strategies, harm-reduction principles, and human-rights-based approaches, and intervention fidelity was monitored through direct observation by trained research staff using structured rating forms to assess adherence and participatory delivery.

### 2.6 Ethical considerations

All procedures were approved by the Institutional Review Board of the University of Texas at El Paso and written informed consent was obtained from all CHAs and participants before their participation in training or intervention activities. Because the peer-network sessions covered HIV prevention, sexual health, and harm reduction related to substance use in small groups, we were aware that participants might feel pressured to share personal HIV-related information. Sharing HIV status, testing history, sexual practices, or substance-use experiences was not mandatory for participation. At each session’s start, Peer CHAs reviewed rules about respect, confidentiality, and participants’ right to skip questions or sharing. The activities emphasized general risk-reduction knowledge and skills over personal HIV disclosures. Participants with concerns or needing individualized support were offered private follow-up with project staff or partner-organization personnel and referrals to appropriate HIV testing, treatment, and harm-reduction services. CHAs were trained in research ethics, confidentiality, trauma-informed engagement, and crisis response, including managing suicide risk, acute intoxication, and exposure to violence. Study data were stored in secure, password-protected systems, with personal identifiers maintained separately from de-identified analytic datasets, and participants were regularly reminded that their participation was voluntary and could be discontinued at any time without affecting access to services. All participants received up-to-date information on local resources for HIV/HCV testing, harm-reduction services, substance-use treatment, and social support.

### 2.7 Fidelity monitoring

Across both El Paso and Ciudad Juárez, CHAs demonstrated high proficiency in delivering all components of the peer-led behavioral intervention. By the end of implementation, CHAs trained a total of 300 participants, 98% of whom attended all sessions within their assigned cohort.

Intervention sessions were directly observed by trained research staff using a structured fidelity assessment tool to evaluate whether each activity achieved its intended objectives. Each session comprised seven to nine discrete activities, and observers rated each activity on a three-point scale (2 = objective met without modification; 1 = objective met with modification; 0 = objective not met). Observers also provided written qualitative notes to substantiate each rating. Activity scores were summed within sessions, with total possible scores ranging from 14 to 18, and divided by the maximum attainable score to generate a session-level fidelity rating. Weekly meetings with peer leaders and observers were conducted to review implementation challenges and identify corrective strategies. The fidelity rating was 90%.

### 2.8 Impact of intervention on peer influence and sexual risk behavior

**Figure 1:**
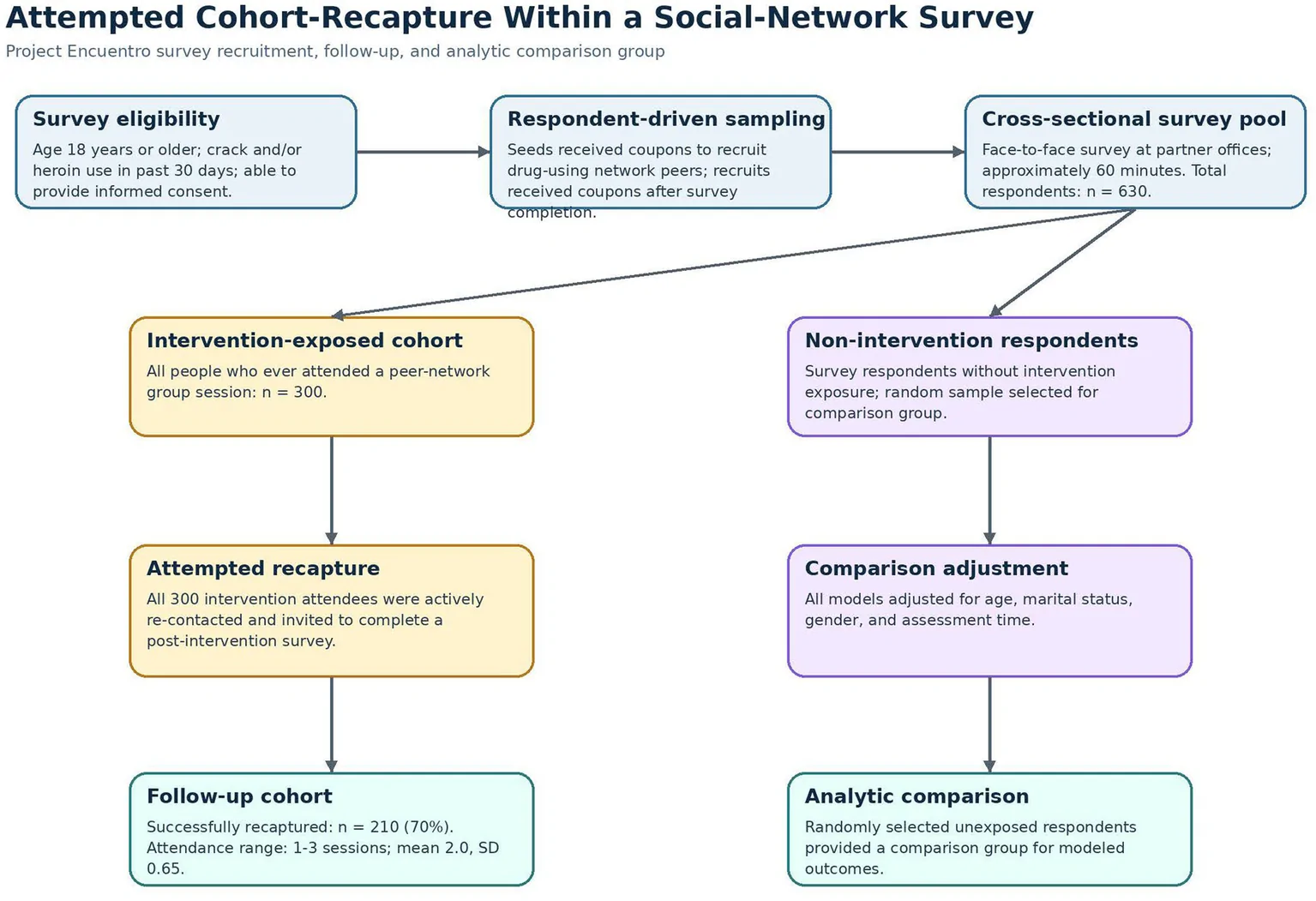
Attempted cohort-recapture design nested within the Project Encuentro social-network survey. All individuals who had attended at least one peer-network group session (*n* = 300) were actively re-contacted for post-intervention assessment; 210 participants were successfully recaptured (70%). The broader respondent-driven social-network survey included 630 eligible respondents, from whom a random sample of non-intervention respondents was selected as the comparison group. Models adjusted for age, marital status, gender, and assessment time.

We used an attempted cohort-recapture design nested within a broader social-network survey. All 300 individuals who had ever attended a peer network group session were actively re-contacted and invited to complete a post-intervention survey; 210 were successfully recaptured and provided follow-up data. Among those who initiated the intervention, attendance ranged from 1 to 3 sessions, with a mean of 2 (SD = 0.65) sessions attended. Because recruitment for Project Encuentro used social-network methods, survey participation was open to people regardless of intervention exposure, allowing us to draw from 630 total survey respondents to construct a comparison group. Among the non-intervention respondents, we selected a random sample and, in all models, adjusted for age, marital status, gender, and assessment time to reduce confounding from demographic differences and secular trends (Figure 1).

Survey eligibility required being at least 18 years old, having used crack and/or heroin in the past 30 days, and being able to provide informed consent. Eligibility was restricted to adults reporting recent crack and/or heroin use because the intervention was designed for drug-using social networks whose primary drug was crack and/or heroin, which research identifies as settings with elevated sexual and drug-related HIV risk and dense peer ties through which prevention messages can diffuse. Participants were recruited using respondent-driven sampling chains initiated by a small number of seeds, each given coupons to invite members of their drug-using social networks; each new participant who completed the survey then received coupons to recruit additional peers, with coupon codes allowing tracking of recruitment and survey participation over time. Surveys were administered face-to-face in private spaces at partner organizations’ offices, lasted about 60 min, and participants received $5.00 for participation and $5.00 for each eligible peer they recruited. These amounts were set in consultation with the CAB and deemed as consistent with local pay rates while minimizing potential coercion.

### 2.9 Measures

A systematic translation and back-translation process was employed to ensure English-Spanish language equivalence of intervention manuals and measures. A community advisory board engaged in the following 3-step translation process including a: (1) forward translation (English to Spanish), back translation (Spanish to English by another translator); (2) meetings to review and resolve discrepancies between the versions by consensus.

Survey questions assessed city of residence, gender, age, education, income, and marital status. Substance use measures assessed lifetime and age of first use of alcohol, heroin, and crack, plus days used in the past 30, and number of times used yesterday for each substance. Cross-border use was captured with an item asking whether they had crossed the border in either direction to use drugs (0 = ‘no’; 1 = ‘yes’). The outcome measures were condom and sanitized syringe procurement, and condomless sex. Condom procurement was assessed by asking the frequency with which participants bought and carried condoms (0 = ‘never’ to 5 = ‘always’) (inter-item correlation = 0.38). As an indicator of safer injection practices, the survey assessed syringe procurement by asking the frequency with which they bought and carried sanitized syringes (0 = ‘never’ to 5 = ‘always’) (inter-item correlation = 0.43). Condomless sex was assessed by asking participants to report the number of sex acts with substance-using partners and the number of those acts without a condom. These two variables were divided to create the proportion of condomless sex acts with high-risk substance using partners.

The following peer processes were assessed: *Peer shared HIV prevention information* with an eight-item checklist asking, “What HIV/AIDS prevention topics did you receive from peers?” Participants indicated yes/no for information about: (1) risk behaviors in their social network, (2) HIV testing, (3) the relationship between drug use and HIV risk, (4) situations that increase risk of acquiring or transmitting HIV, (5) sexually transmitted infections, (6) sexual rights, (7) services that can help reduce or stop drug use, and (8) sexual behaviors that increase HIV risk. Items were coded (0 = ‘no’; 1 = ‘yes’) and summed, with higher scores indicating exposure to a broader range of HIV-related prevention content. Peer shared HIV prevention information captures individual participants’ self-reported exposure across topics and sessions. Variation in attendance, engagement, and recall meant that participants differed in how much information they reported receiving. *Peer behavior change* was assessed with three yes/no items asking whether, in the past six months, participants had (1) used condoms, (2) obtained an HIV test, and (3) used harm reduction services because of the intervention sessions. We evaluated the receipt of harm-reduction services in relation to peer-network sessions because these services, such as condoms, sterile injection equipment, were directly integrated into and reinforced during the intervention. Other service items were not included in the peer behavior-change index, as they could be accessed from various organizations and were not directly linked to peer-network session participation. The index was thus designed to reflect behaviors and resources most likely associated with the intervention’s peer-delivered harm reduction content, rather than general health or social service use. Items were coded 1/0 and summed to create a peer behavior change index, with higher scores indicating a greater number of concrete HIV-preventive behaviors attributed to the workshops. The independent variable was participation in the peer-led intervention: (0 = ‘did not participate in the peer-led intervention’; 1 = ‘participated in the intervention.’). We hypothesized that peer behavior change would be associated with reductions in condomless sex and that peer-shared HIV prevention information would be a mediator of this association. Condomless sex was specified *a priori* in the parent trial as the primary HIV-related risk outcome, with injection-related behaviors designated as secondary outcomes. Consistent with this design, the present mechanistic analysis focuses on the primary sexual risk outcome.

### 2.10 Statistical analysis

Survey administration by trained interviewers led to a low item nonresponse rate (<5% for all variables used in analyses). Because missing data was minimal, simple imputation was used to replace missing values in the dataset. We analyzed intervention effects on three primary outcomes, condom procurement, procuring new or sanitized needles, and condomless sex, using generalized linear models estimated in IBM SPSS Statistics (version 29.0). For the condom and syringe procurement outcomes (continuous frequency of carrying and procuring), we fit a linear model with normal distribution and identity link, including intervention status as the main predictor and adjusting for assessment wave, recent heroin use, and age, while clustering standard errors by recruiter to account for non-independence among participants recruited through the same social-network chains. For the proportion of sex acts with substance use partners that occurred without a condom, we used generalized linear models with appropriate link functions, again including intervention status as the primary exposure and controlling for age, gender, income, marital status, assessment wave, participation in other intervention components, and recruiter clustering. Robust (sandwich) covariance estimates were used in all models, Wald chi-square tests assessed the significance of predictors, and adjusted marginal means with 95% confidence intervals were computed to aid interpretation of intervention effects on each outcome.

We tested a path model to examine whether receipt of HIV prevention information mediated the association between peer behavior change and condomless sex. We tested the model in Mplus with maximum likelihood estimation and a complex survey specification (clustering on recruiter), we regressed the continuous indicator of condomless sex on receipt of peer information, the peer behavior change index, and covariates (age, gender, marital status, and assessment wave), and simultaneously regressed peer information on peer behavior change and the same covariates. This specification allowed us to estimate direct, indirect, and total effects of peer behavior change on condomless sex via peer information, with 5,000 bias-corrected bootstrap draws to obtain confidence intervals for indirect effects.

### 2.11 Qualitative data collection and analysis

Upon completion of the study, CHAs were invited to participate in in-depth, semi-structured interviews to share their perspectives on the intervention’s effects on participants’ harm-reduction and sexual-risk behaviors. The semi-structured interview guide covered topics such as CHAs’ experiences with training and supervision, their changing roles, the perceived impact of the intervention on participants’ injection and sexual risk behaviors, peer communication and information sharing. Interviews were audio-recorded with consent, transcribed verbatim in the original language, and analyzed using a rapid qualitative approach. We used a team-based rapid qualitative analysis approach to generate timely, practice-relevant findings about CHA experiences and intervention implementation. Rapid qualitative analysis uses structured templates and matrices to systematically condense interview data while preserving attention to context and cross-participant variation (29, 30). Rather than conducting line-by-line coding of all transcripts, trained coders independently reviewed each transcript and completed a structured summary template organized around prespecified domains of interest: CHA training and support; role identity and professional development; perceived effects on participants; peer communication and information diffusion; and gendered barriers to intervention delivery and engagement. Coders also recorded unanticipated or emergent issues that did not fit within the initial domains.

Completed summaries were entered into analytic matrices organized by participant and analytic domain. These matrices facilitated comparison across CHAs, identification of convergent and divergent experiences, and iterative refinement of domain definitions. The research team met regularly to review summaries, discuss interpretive differences, reconcile discrepancies through consensus, and identify patterns that were meaningful across cases. Themes were defined as recurring, conceptually coherent patterns across participants’ accounts that addressed the study aims; they were developed iteratively from both the prespecified domains and emergent observations (31).

Qualitative findings were analyzed as a complementary source of evidence rather than as a formal test of quantitative results. During interpretation, the team considered how CHA accounts contextualized quantitative findings, particularly those related to peer discussions, receipt and dissemination of HIV-prevention information, and perceived peer behavior change as potential pathways of intervention impact.

## 3 Results

### 3.1 Quantitative analyses

Participants were 418 people who use drugs, 30.4% female, 0.2% transgender male to female, with a mean age of 42.73 years (SD = 11.78, range = 18–70). Participants were recruited from two cities along the U. S.-Mexico border: Ciudad Juárez, Mexico (52.9%) and El Paso, Texas (47.1%). Educational attainment was low overall, with the majority (74.8%) reporting less than high school completion. Just over half of participants identified as single (52.2%), while 29.1% were married or living with a partner. Participants reported highly variable income (M = 345.17, SD = 617.42), with one-third (33%) indicating no income, reflecting substantial economic marginalization. Substance use profiles indicated 98% had injected heroin and used crack in the past 30 days, early initiation of alcohol and subsequent progression to heroin and crack, with 96.7% reporting lifetime alcohol use (mean age of first use 13.6 years), 90.7% lifetime heroin use (mean initiation age 18.9 years), and 90.9% lifetime crack use (mean initiation age 20.9 years), and with 52.8% reporting daily heroin use and 28.8% reporting daily crack use in the past 30 days. Overall, 38.8% of PWUD reported having crossed between El Paso and Ciudad Juárez to consume drugs in the last 30 days.

Results of the GLM revealed that the intervention showed protective effects on sexual risk but only modest effects on sanitized syringe procurement. Participation in the intervention was associated with greater condom procurement; participants exposed to the intervention reported higher adjusted mean levels of obtaining condoms 2.60 than non-participants 2.23 (95% CIs 2.20—3.0 and 1.90–2.56, respectively; B = 0.36, Wald χ^2^ = 3.99, *p* = 0.046), reflecting a small-to-moderate protective effect on safe sex (standardized difference ≈ 0.20), indicating increased engagement with HIV prevention resources. In contrast, intervention participation was associated with a small but statistically significant reduction in the frequency of carrying and purchasing sanitized syringes: adjusted mean 1.46 episodes for intervention participants versus 1.66 for non-participants (95% CIs 1.24–1.68 and 1.46–1.86, respectively; B = 0.20 favoring the non-intervention group, Wald χ^2^ = 3.92, *p* = 0.048), corresponding to a small effect size (standardized difference ≈ 0.16). For sexual behavior, intervention participants reported a significantly lower proportion of condomless sex than those not exposed—adjusted means 0.42 vs. 0.51 (95% CIs 0.34–0.51 and 0.43–0.61, respectively; B = −0.20, Wald χ^2^ = 4.64, *p* = 0.031), reflecting a small-to-moderate protective effect on sexual risk (standardized difference ≈ 0.20). Overall, these findings suggest that the peer-network intervention was more successful at shifting sexual-risk behaviors and condom-related prevention practices.

Results indicated that greater peer behavior change was strongly associated with receiving more HIV-related information (standardized *β* = 0.53, *p* < 0.001), and that receiving more information, in turn, was associated with a lower level of condomless sex (standardized *β* = −0.18, *p* = 0.012), adjusting for age, gender, and assessment wave. Peer behavior change was not directly related to condomless sex (standardized *β* ≈ 0.00, *p* = 0.99) but exerted a significant indirect effect through peer information (standardized indirect effect = −0.10, *p* = 0.012), yielding a non-significant total effect. Peer information accounted for 36% of the variance (R^2^ = 0.36), and the model explained approximately 10% of the variance in condomless sex (R^2^ = 0.10). Taken together, these findings support a mediated pathway in which peer-attributed behavior change relates to reduced condomless sex primarily through increased exposure to HIV prevention information, rather than via a direct effect on sexual risk.

### 3.2 Qualitative results

The mean age of CHA, 50% female, was 39.75 years (SD ≈ 6.83, range = 32–48). Rapid qualitative analysis identified three interconnected themes illustrating CHAs’ perceptions of how the intervention was implemented and its effects. Throughout the interviews, CHAs highlighted that prevention information was most effectively received and shared when delivered by trusted peers, made practical through interactive group activities, and supported by organizational structures that address participants’ social and gender vulnerabilities. These themes offer valuable context for understanding the quantitative data on peer discussions, HIV-prevention information receipt, and perceived peer behavior changes. A matrix-based comparison of structured interview summaries revealed three interconnected, implementation-focused themes: (1) trusted peer relationships that facilitated the adoption and spread of prevention information; (2) interactive delivery methods that translated prevention content into practical harm-reduction skills; and (3) organizational support coupled with gender-responsive practices influencing CHA delivery. Collectively, these themes provide context for the quantitative data on peer discussions, the receipt and dissemination of HIV-prevention information, and perceived changes in peer behavior.

### 3.3 Theme 1: trusted peer relationships enabled uptake and diffusion

CHAs described information diffusion as a relational process rather than a one-way transfer of HIV-prevention messages. Participants were more willing to consider and share harm-reduction information when it came from peers with lived experience and from an organization they regarded as trustworthy. In this account, trust was not simply a background condition for participation; it was the mechanism that enabled prevention information to be received, discussed, and transmitted through drug-using networks.

For injection risk, one CHA explained:

"*So, through this information many of them began to tell us that they preferred to wait ten more minutes and be able to get a syringe, rather than going to an abandoned building and looking for one they found lying around. This information helped these people a lot and, also, they themselves passed it on to other people*."

This account suggests that participants described moving from immediate, high-risk syringe sharing reuse toward more deliberate efforts to obtain sterile equipment. The CHA also described participants as sharing this information with others, suggesting a peer-diffusion process that is consistent with the quantitative association between peer-shared prevention information and lower reported risk behavior.

CHAs’ shared language, lived experience, and affiliation with trusted community organizations fostered relationships that made prevention messages believable and acceptable to participants. One CHA remarked:

"*This training they gave us and the way we passed it on to them was what really opened the way for them to be willing to enact prevention behaviors like taking an HIV test*."

This quote illustrates that the training CHAs received, and their ability to transmit it authentically, “opened the way” for participants to accept HIV testing, emphasizing that credibility stemmed from both personal experience and formal preparation.

Another CHA described how organizational reputation fostered confidence:

"*I think the seriousness of the program… when they hear that it is from Compañeros, it is like a synonym for trust, that it is something good for them, that nothing bad is going to happen to them*."

Here, the CHA underscores that Programa Compañeros’ established reputation served as a “synonym for trust,” reassuring participants that the intervention was safe and beneficial, not harmful. This organizational credibility combined with CHAs’ relatability (“one of them”) to create conditions under which prevention information could be received and acted upon.

Collectively, these accounts indicate that CHA credibility stemmed from lived experience, formal intervention training, and the well-established reputation of Programa Compañeros. This credibility seemed to make prevention information more practical and enabled participants to act as secondary messengers within their networks. These insights contextualize the quantitative link between peer-shared HIV prevention information and decreased risk behavior.

### 3.4 Theme 2: interactive delivery made risk reduction feasible

CHAs described the group sessions as most effective when they went beyond simply providing information and instead let participants practice specific harm-reduction techniques. Interactive activities, especially role plays centered on condom negotiation, helped participants think about how to handle pressure, power imbalances, and risks in real-life situations.

One CHA noted:

"*Well, there were parts where we did some activities so that the women could negotiate using a condom… they played roles of people who, for example, would go with them and try to get them not to use a condom and how to negotiate. So, seeing them acting it out helped people a lot because that way they saw it as more visible, more manageable*."

This quote highlights how interactive role-play activities helped participants, especially women, practice condom negotiation in realistic scenarios. Seeing peers enact these situations made safer-sex strategies feel “more visible, more manageable,” allowing participants to rehearse negotiation skills in a supportive environment. This peer-led modeling made abstract concepts tangible and applicable to real-world situations, aligning with quantitative evidence that increased peer discussion and receipt of HIV prevention information, rather than large immediate shifts in behavior, underpinned reductions in condomless sex with crack-using partners.

CHAs outlined a similar approach to reducing injection-related harm. Group discussions prompted participants to reflect on recent behaviors, such as sharing syringes and pipes, and to think about small, achievable modifications in their drug-use environments. One CHA mentioned asking participants if they had altered their syringe-sharing or condom-use practices after the sessions.

“*For example, when we had these discussions with people who use drugs, there came a point when I would ask them, in the most recent days they had attended, who had stopped sharing syringes and who had started using condoms. Most of them said that, through the information we gave them, they had become aware that they did not need to keep lending their syringes. I would say that around 60% of those people were very motivated and stopped sharing syringes or pipes with one another, which can also transmit infections.*”

The CHA’s account indicates that the sessions served as a regular platform where participants could relate prevention information to their recent actions and openly acknowledge their efforts to change. The CHA’s reference to “around 60%” reflects an informal observation rather than a formal estimate of the intervention’s effectiveness. This account suggests that the sessions created a recurring space for participants to reflect on recent risk behaviors, discuss feasible alternatives, and recognize attempts to reduce syringe sharing.

Across sexual and injection-related risk areas, CHAs highlighted the importance of providing participants with chances to practice applying prevention strategies in real-life drug use and sexual decision-making. Techniques such as role play, guided reflection, and peer discussions helped turn broad HIV prevention messages into concrete actions, which participants saw as tangible, achievable, and relevant to their local situations. This pattern may clarify why participant-reported receiving HIV-prevention information and engaging in peer discussions were associated with reported behavior change: the intervention combined information with peer modeling, practice, and discussion of strategies that are locally feasible, rather than relying solely on didactic information.

### 3.5 Theme 3: support and gender-responsive practice shaped delivery

CHAs described their ability to deliver the intervention as dependent on continuing organizational support and the capacity to respond flexibly to participants’ emotional, gendered, and stigma-related concerns. Their roles extended beyond delivering a curriculum to include facilitating difficult conversations, recognizing distress, protecting privacy, and connecting participants to additional support.

One CHA reflected on the institutional support received:

"*Well, in reality, (Programa Compañeros) were always supporting me, sending me reminders and all those kinds of things. So I always felt well supported by them*."

This statement underscores that consistent organizational support, reminders, supervision, and guidance, enabled CHAs to feel “well supported” throughout the intervention, which was critical to their ability to facilitate groups, manage conflict, and address sensitive topics such as stigma, trauma, and HIV status.

Another CHA described how implementing the intervention strengthened her own learning and sense of protection:

"*Because I learned a lot from my peers… with me being… a drug user, I would think, ‘Wow!’… as if many times I was saved. Protected so that not as much happened to me as has happened to them*."

Here, the CHA reveals that she internalized prevention messages herself while delivering them to others, feeling “saved” and “protected” from harms that her peers had experienced. This dual role, as both learner and teacher, both beneficiary and protector, illustrates how the capacity-building model generated benefits beyond immediate client-level risk reduction and sustained CHAs’ motivation to initiate conversations, share information, and model safer behaviors. Some CHAs described increased confidence and expanded responsibilities in outreach, peer support, and navigation following their participation in the intervention.

This theme addresses gender-specific barriers to HIV testing and risk disclosure, particularly among women, and the strategies CHAs used to create emotionally safe spaces for prevention messages. One CHA recounted an instance in which a participant became distressed during a group session. The CHA indicated that “She stopped the group and took her outside” to talk privately and connect her to testing “so that the other people wouldn’t find out,” emphasizing the importance of protecting participants from stigma. This narrative illustrates that CHAs recognized when group settings became unsafe and actively managed emotional distress by pausing activities and providing individual support discreetly, ensuring that other participants did not become aware of the sensitive issue. This tactful approach protected confidentiality and dignity. CHAs also described fear of a positive HIV test result and anticipated stigma as barriers that could discourage women from testing or discussing risk. These accounts indicate that gender-responsive delivery required more than including women in group activities; it required attention to privacy, emotional safety, and the potential consequences participants associated with disclosure.

These accounts illustrate that fidelity to intervention content alone was insufficient for effective delivery. CHAs required ongoing supervision and institutional backing to manage unanticipated crises and adapt sessions without compromising participants’ dignity or confidentiality. Gender-responsive engagement was particularly important because women’s fear of testing, anticipated stigma, and concerns about disclosure could limit participation in HIV-prevention activities.

CHAs also described reciprocal benefits of their roles: delivering the intervention reinforced their own harm-reduction knowledge, strengthened their confidence, and supported progression from outreach roles toward leadership, navigation, mentoring, and recovery-oriented work. This suggests that peer capacity building was both an implementation strategy and an outcome of the intervention.

Across themes, CHAs portrayed intervention implementation as dependent on the interaction of trusted peer relationships, practical skill-building, and organizational support responsive to participants’ safety and disclosure concerns. These accounts contextualize how peer-delivered HIV-prevention information may have functioned as a pathway to reported harm-reduction behavior change.

## 4 Discussion

This study suggests that peer-led, network-based HIV prevention may influence sexual risks among people who use crack and inject drugs, potentially through enhanced peer communication and information diffusion. Quantitative analyses showed intervention participation was associated with increased condom procurement and significantly lower condomless sex. Path models indicated that peer-shared prevention information was associated with reduced condomless sex, with peer behavior change exerting an indirect association through increased information flow rather than through a direct pathway. CHA accounts provided implementation-focused context for these quantitative associations. Across the qualitative themes, CHAs described howtrusted peer relationships and organizational credibility increased the acceptability of prevention messages; how role plays, discussions, and peer modeling helped translate prevention content into practical actions; and how organizational support and gender-responsive practices enabled CHAs to address issues like distress, stigma, privacy, and disclosure during intervention delivery. These qualitative insights shed light on potential processes by which peer-delivered information was received, practiced, and shared within drug-using networks.

These findings align with systematic reviews showing that peer education produces sustained but moderate improvements in HIV risk behaviors (32). Our observation that peer communication and information sharing were associated with intervention-related changes parallels peer interventions in Baltimore and elsewhere that have documented network-level reductions mediated by knowledge mechanisms (33, 34). Recent analysis from HPTN 037 showed that recall of intervention knowledge mediated effects of a peer-driven intervention on injection behaviors among people who inject drugs (35), providing a relevant comparison for our finding that peer-shared information was associated with condomless sex. While much peer HIV-prevention research focuses on behavioral outcomes, the present path models and CHA accounts are consistent with information diffusion through trusted peer channels as a plausible pathway linking peer intervention processes with individual risk-reduction behavior.

Our results support the expanding evidence for network-based HIV prevention among PWUD. Research in Vietnam and India has linked network norms, peer support, and engagement with prevention programs (36, 37), but fewer studies have explored prevention information as a mediator between peer influence and sexual-risk behaviors. In this study, the path models indicated that peer-attributed behavior change influenced condomless sex indirectly via information sharing. This aligns with diffusion-of-innovations theories, which suggest that credible communication within tight-knit social networks can promote the adoption of new practices (38). The qualitative themes provide practical insights: CHAs highlighted trust and organizational credibility as key factors for message acceptance, while role plays and group discussions helped participants incorporate prevention information into condom negotiation, syringe access, and other urgent decisions. The qualitative findings also underscore that effective peer delivery required more than accurate information or strong peer relationships. CHAs described relying on organizational support, supervision, and flexibility to address participants’ emotional distress, protect privacy, and respond to gendered barriers to HIV testing and risk disclosure. In particular, accounts of pausing group activities to speak privately with distressed participants suggest that confidentiality and emotional safety were integral implementation conditions, not peripheral concerns. These findings indicate that peer-led interventions may be most effective when CHAs are supported by organizations capable of providing supervision, referral pathways, and flexible responses to stigma, violence, and competing survival needs.

Findings also point to the broader structural and emotional constraints that can temper the impact of peer interventions, even when information is accurate and relationships are strong. CHAs described working with participants, particularly women, who feared HIV test results and potential stigma, sometimes needing to pause groups and move conversations to more private settings so that “other people would not find out,” suggesting that concerns about safety, judgment, and immediate survival can take precedence over risk reduction. These constraints are consistent with qualitative research from West African harm-reduction initiatives, where peers successfully shift norms but cannot fully counteract poverty, policing, or housing insecurity (39). Recognizing these realities, future interventions must combine peer capacity-building with structural supports, including expanded syringe access, safe consumption sites, income generation, and legal protection for PWUD.

### 4.1 Limitations

This study has several limitations that should be considered when interpreting the findings. First, the quasi-experimental design and reliance on self-reported measures preclude definitive causal inference; although the path models are consistent with an information-diffusion mechanism, unmeasured confounding and reverse causation remain plausible explanations. Second, key mediators and outcomes, peer-attributed behavior change, receipt of HIV prevention information, and condomless sex, were assessed with self-report items increasing the potential for social desirability. Third, the sample size for the mediation analysis (N = 269) and the modest proportion of variance explained in condomless sex suggest that we had limited power to detect small effects and that important determinants of sexual risk, including structural and relational constraints, were not captured by our models. Fourth, our mechanistic analysis focused on condomless sex with substance-using partners and did not extend parallel models to injection-related outcomes or transactional sex, which may limit inferences about how broadly the observed mechanism applies across different HIV risk domains. Fifth, attrition over time and incomplete follow-up for some participants raise the possibility of attrition bias, particularly if individuals with the highest levels of structural vulnerability were less likely to be retained.

Finally, the study was conducted among Latine PWUD in a single binational metropolitan area with a specific harm-reduction infrastructure and long-standing community–academic partnerships, which may limit generalizability to non-border settings or populations with different demographic and drug-use profiles; replication in diverse contexts and with longitudinal designs is needed to more firmly establish the robustness and transportability of the mechanisms identified here. At the same time, the study contributes novel evidence on the feasibility and added value of investing in CHA capacity building within an established harm-reduction infrastructure. By documenting CHAs’ progression from traditional outreach workers to skilled peer interventionists, navigators, and recovery coaches, the findings highlight how structured training and supervision can enhance peers’ confidence, leadership, and ability to deliver complex, mechanism-focused interventions. These strengths suggest that, despite the noted design and measurement limitations, the study offers a useful proof-of-concept for peer-led models that explicitly target information diffusion pathways and CHA role development in highly marginalized border communities.

### 4.2 Future directions

Future research should test network-based peer interventions with stronger longitudinal designs and explicit causal mediation frameworks. Cohort studies that repeatedly measure peer communication, information exchange, and behavior over multiple time points could clarify the temporal ordering of these processes and provide more definitive evidence for causality. Combining sociometric network mapping with intervention data could identify which CHA characteristics and network positions maximize information diffusion and behavior change uptake. Given the structural barriers highlighted by our qualitative findings, future interventions should consider testing combinations of peer components with expanded harm-reduction infrastructure (e.g., safer consumption sites, syringe vending machines), legal protections for PWUD, and economic supports (e.g., conditional cash transfers, employment programs).

## 5 Conclusion

This study provided evidence that a peer-led, network-based intervention was associated with lower condomless sex and greater condom-related prevention, with peer-shared information emerging as the central mechanism linking peer behavior change to sexual risk reduction. Integration of quantitative path models and qualitative accounts from CHAs supports a diffusion-of-innovations interpretation in which CHAs’ conversations and modeling seed risk-reduction practices that spread through networks, even amid structural constraints. These findings underscore the value of investing in peer capacity and network-aware strategies as part of comprehensive HIV risk reduction in marginalized drug-using communities, while also calling for structural reforms that enable peers to act on the information they receive.

## Data Availability

The datasets presented in this study can be found in online repositories. The names of the repository/repositories and accession number(s) can be found at: https://datadryad.org/.

## Footnotes

1 Latine is a gender-inclusive term used in some U.S. and Latin American contexts to refer to people of Latin American origin; hereafter ‘Latine’ for consistency. Other terms such as Latino/a and Latinx are used in different regions.
